# Lag Analysis of Fast fMRI Reveals Delayed Information Flow Between the Default Mode and Other Networks in Narcolepsy

**DOI:** 10.1093/texcom/tgaa073

**Published:** 2020-10-10

**Authors:** M Järvelä, V Raatikainen, A Kotila, J Kananen, V Korhonen, L Q Uddin, H Ansakorpi, V Kiviniemi

**Affiliations:** Department of Diagnostic Radiology, Medical Research Center (MRC), Oulu University Hospital, 90220 Oulu, Finland; Research Unit of Medical Imaging, Physics and Technology, The Faculty of Medicine, University of Oulu, 90220 Oulu, Finland; Department of Diagnostic Radiology, Medical Research Center (MRC), Oulu University Hospital, 90220 Oulu, Finland; Research Unit of Medical Imaging, Physics and Technology, The Faculty of Medicine, University of Oulu, 90220 Oulu, Finland; Research Unit of Logopedics, University of Oulu, 90014 Oulu, Finland; Department of Diagnostic Radiology, Medical Research Center (MRC), Oulu University Hospital, 90220 Oulu, Finland; Research Unit of Medical Imaging, Physics and Technology, The Faculty of Medicine, University of Oulu, 90220 Oulu, Finland; Department of Diagnostic Radiology, Medical Research Center (MRC), Oulu University Hospital, 90220 Oulu, Finland; Research Unit of Medical Imaging, Physics and Technology, The Faculty of Medicine, University of Oulu, 90220 Oulu, Finland; Department of Psychology, University of Miami, Coral Gables, 33124 FL, USA; Research Unit of Clinical Neuroscience, Neurology, University of Oulu, 90014 Oulu, Finland; Department of Neurology, Oulu University Hospital, 90014 Oulu, Finland; Department of Diagnostic Radiology, Medical Research Center (MRC), Oulu University Hospital, 90220 Oulu, Finland; Research Unit of Medical Imaging, Physics and Technology, The Faculty of Medicine, University of Oulu, 90220 Oulu, Finland

**Keywords:** default mode network, fast fMRI, information flow, lag analysis, narcolepsy

## Abstract

Narcolepsy is a chronic neurological disease characterized by dysfunction of the hypocretin system in brain causing disruption in the wake-promoting system. In addition to sleep attacks and cataplexy, patients with narcolepsy commonly report cognitive symptoms while objective deficits in sustained attention and executive function have been observed. Prior resting-state functional magnetic resonance imaging (fMRI) studies in narcolepsy have reported decreased inter/intranetwork connectivity regarding the default mode network (DMN). Recently developed fast fMRI data acquisition allows more precise detection of brain signal propagation with a novel dynamic lag analysis. In this study, we used fast fMRI data to analyze dynamics of inter resting-state network (RSN) information signaling between narcolepsy type 1 patients (NT1, *n* = 23) and age- and sex-matched healthy controls (HC, *n* = 23). We investigated dynamic connectivity properties between positive and negative peaks and, furthermore, their anticorrelative (pos-neg) counterparts. The lag distributions were significantly (*P* < 0.005, familywise error rate corrected) altered in 24 RSN pairs in NT1. The DMN was involved in 83% of the altered RSN pairs. We conclude that narcolepsy type 1 is characterized with delayed and monotonic inter-RSN information flow especially involving anticorrelations, which are known to be characteristic behavior of the DMN regarding neurocognition.

## Introduction

Narcolepsy is a chronic neurological disease with 2 differentiated phenotypes and a prevalence of 1/2000 (Scammell 2015; Kornum et al. 2017; Sarkanen et al. 2018). Autoimmunity is thought to underlie large proportion of narcolepsy cases, while other etiologies are rare (Mahoney et al. 2019). Narcolepsy type 1 is caused by loss of neuropeptide, hypocretin, producing cells in the posterolateral hypothalamus that project throughout the cortex to promote wakefulness and cortical excitation (Scammell 2015; Bassetti et al. 2019). The typical symptoms include daytime sleepiness with sleep attacks and cataplexy characterized as transient loss of muscle tone triggered by emotional stimuli. Narcolepsy type 2 is a less severe form of the condition without cataplexy.

Moreover, clinically relevant cognitive symptoms are common in narcolepsy type 1 (Fulda and Schulz 2001). Earlier studies on attention have shown impairment in monotonous and long tasks in narcolepsy (Valley and Broughton 1981; Godbout and Montplaisir 1986). Electroencephalography studies suggest prolonged information processing and alterations in cognitive preattentive and attentive processes characterized by prolonged auditory/visual event-related potential component N2 and P300 latencies with increased P300 amplitude in narcolepsy (Sangal et al. 1999; Naumann et al. 2001; Saletu et al. 2008). A study utilizing visual discrimination task found that patients with narcolepsy had worse performance than healthy controls in training and in 2 delayed retrieval sessions indicating lower level of visual skill consolidation (Cipolli et al. 2009). The same experimental design revealed worse performance and reduced positive change in performance across sessions in narcolepsy when investigating procedural motor skill consolidation (Mazzetti et al. 2012) suggesting initial lower encoding level (Cellini 2017). Furthermore, patients with narcolepsy have shown generally slower and more varied responses in cognitive tasks with longer reaction times compared with healthy controls (Rieger et al. 2003; Bayard et al. 2012). Taken together, a deficient sustained attention and a general dysexecutive profile with proposed misallocation and reduction of available cognitive resources leading to inefficient cognitive control processes has been suggested (Naumann and Daum 2003; Naumann et al. 2006; Witt et al. 2018).

The default mode network (DMN) is most active during wakeful rest, while externally oriented tasks tend to suppress its activity. DMN activity is anticorrelated with task-positive networks including the salience network (SN), dorsal attention network (DAN), and central executive network (CEN) (Fox et al. 2005; Fransson 2005; Chen et al. 2013). These networks are thought to operate hierarchically and causally in cooperation to facilitate appropriate behavior (Sridharan et al. 2008; Menon 2011; Uddin 2015; Chand and Dhamala 2016; Zhou et al. 2018). Although structural and functional changes have been reported in task- and rest-relevant brain areas in narcolepsy (see Wada et al. 2019 for a review), few resting-state functional magnetic resonance imaging (rs-fMRI) studies have been conducted. These recent investigations into resting-state network (RSN) connectivity in narcolepsy have revealed abnormal brain dynamics, as the patients with narcolepsy were less likely to spend time in an EEG-derived microstate related to the DMN and had similar but not identical mappings of the microstates compared with healthy controls (Drissi et al. 2016). Moreover, within the SN, increased fractional amplitude of low-frequency fluctuation has been reported in narcolepsy along with decreased functional connectivity in both the SN and an executive network and, furthermore, decreased functional connectivity between the DMN and SN/limbic system measured with combined graph theoretical and independent component analysis (ICA) (Xiao et al. 2018, 2019, 2020).

Most prior fMRI studies of spontaneous brain activity have utilized either spatial ICA (sICA) (Kiviniemi et al. 2003; Beckmann et al. 2005) or seed-based correlation mapping (Biswal et al. 1995, 2010) to chart functional networks. Critically, these analyses assume that activity within RSNs is exactly synchronous, that is, zero-lag connectivity. However, recent rs-fMRI studies in humans and rats suggest that spontaneous brain activity is spatiotemporally structured (Majeed et al. 2009, 2011; Chang and Glover 2010; Kiviniemi et al. 2011; Hutchison et al. 2013; Liu and Duyn 2013), and that multiple temporal functional modes in human rs-fMRI data exist (Smith et al. 2012; Raatikainen et al. 2017).

Temporal lags reflect a time delay in brain activation propagation between brain areas. Some regions are early (sources of propagation) and some regions are late (destinations of propagation) (Mitra et al. 2014; Mitra, Snyder, Blazey, et al. 2015). The lag structure of rs-fMRI is highly reproducible (Mitra, Snyder, Blazey, et al. 2015; Raut et al. 2019), and it has been shown that these lag-related propagation patterns are altered as a function of state, whether pathological (Mitra, Snyder, Constantino, et al. 2017; Shah et al. 2018; Bandt et al. 2019; Raatikainen et al. 2019) or physiological (Mitra, Snyder, Tagliazucchi, et al. 2015). Thus, propagation can be a more sensitive marker in some pathologies than conventional functional connectivity analysis (Mitra, Snyder, Constantino, et al. 2017). However, as lag analyses greatly benefit from high temporal resolution, there is a need for faster data acquisition than possible with conventional fMRI (Lin et al. 2013; Mitra, Snyder, Blazey, et al. 2015; Rajna et al. 2015; Raatikainen et al. 2017; Huotari et al. 2019). A recently described dynamic lag analysis (DLA) approach, together with critically sampled data, measures inter-RSN time lag variations and statistically defines how the lag patterns are altered between study groups (Raatikainen et al. 2019). Instead of assuming a single temporal lag over a time epoch as in cross-correlation-based analysis (e.g*.*, Mitra et al. 2014; Mitra, Snyder, Blazey, et al. 2015; Mitra, Snyder, Constantino, et al. 2017), the DLA approach determines time lags, peak-by-peak, over the whole time series, thus offering analysis independent from correlation calculation between time series with dynamic information on patterns of information flow, that is, how the time lag and directionality vary between RSNs over time. To elaborate, DLA accounts for each peak and nadir of the fMRI time series enabling investigation of dynamic signal behavior between spatial locations in same phased (from positive to positive and from negative to negative) and antiphased (from positive to negative and from negative to positive) configurations that remain obscured in correlation-based analyses, for example, sliding-window approach.

In this study, we utilize the DLA concept and fast fMRI sequence (magnetic resonance encephalography: MREG, TR = 100 ms) imaging to study, for the first time, the temporal fMRI signal propagation patterns between major RSNs in narcolepsy type 1 and healthy controls. We further examine time lags in anticorrelated RSN pairs—when signals are in opposed phases (positive vs. negative peak and vice versa)—in addition to the activated (positive peaks) and deactivated RSNs (negative peaks). The separate analysis of antiphased time signals (positive to negative and negative to positive) enables investigation of the inter-RSN lag variability in detail when an activation of one network leads to deactivation of the other and vice versa. With the suggested dysfunction of intrinsic RSN dynamics and cognitive deficits described in the previous research, we hypothesize that (1) a dynamic inter-RSN dysfunction exists in narcolepsy type 1, that (2) it can be accurately quantified with the lag-based DLA, and that (3) these lag pattern variations help to explain the observed cognitive deficits in narcolepsy type 1.

## Materials and Methods

### Participants

A registry run for patients with diagnosis of narcolepsy was conducted from the Oulu University Hospital’s electronic patient records, with 66 matching diagnosis codes found. The diagnoses were based on the codes on the Finnish version of the International Classification of Diseases, 10th edition and the Diagnostic Criteria on the International Classification of Sleep Disorders (ICSD), second/third editions. For this study, all the diagnoses were reassessed with ICSD third edition (American Academy of Sleep Medicine 2014). Twenty-three patients were interviewed via phone and the history of cataplexy as a symptom was confirmed. The inclusion criteria were (1) a confirmed diagnosis of narcolepsy type 1 and (2) cataplexy. The lack of confounding neurological conditions was confirmed by screening the study population for other neurological diseases and brain diseases/trauma. All data were collected between 3/2018 and 3/2019.

Data from 2 patients and their corresponding healthy controls were excluded due to motion during the scan (identified as outliers, see Supplementary Fig. S6). The final population consisted of 21 patients with narcolepsy type 1 (NT1, 12 females, age 28.14 ± 9.16) with 2 unmedicated patients and 19 medically treated for daytime sleepiness, cataplexy, and/or sleep disturbances (Table 1). Twenty-three healthy age- and sex-matched controls (HC) with no continuous medication were recruited from general population, and 21 were used as a control group for this study (12 females, age 28.33 ± 9.22). A written informed consent was obtained from the participants. The study was approved by the Ethical Committee of Medical Research in the Northern Ostrobothnia District of Finland and was conducted in accordance with the declaration of Helsinki with latest GDPR regulations taken into account.

**Table 1:** Patients with narcolepsy type 1

**Subject**	**Sex**	**Age (y)**	**Duration (y)**	**Medication**
1	F	31	3	Mo, SSRI
2	F	40	5	Mo, Me
3	F	27	3	—
4	M	23	1	Mo
5	M	51	16	Mo
6	F	23	9	S
7	F	23	9	Me
8	F	35	3	S, SNRI
9	F	20	2	Me, S
10^¤^	M	27	0	—
11	F	34	6	—
12	M	24	4	Mo
13	F	23	7	Mo, Me
14	F	20	7	Me
15	F	28	8	Me, S
16	F	21	2	Mo, SNRI
17	M	19	4	Mo
18	M	46	4	Mo, S, SNRI
19	M	17	7	Me
20^¤^	M	17	8	Me
21	M	33	4	—
22	M	32	0	Mo
23	M	21	8	Me, S

*Notes:* F = female, M = male, ^¤^ = excluded for motion, Mo = modafinil, Me = methylphenidate, S = sodium oxybate, SSRI = selective serotonin reuptake inhibitor, SNRI = Serotonin–norepinephrine reuptake inhibitor, Age (y) = age in years at time of imaging, Duration (y) = disease duration in years at time of imaging.

### Measurements

All subjects were scanned with fast fMRI sequence called MREG using a Siemens Magnetom Skyra 3 T MRI scanner (Siemens Healthineers, Germany) with a 32-channel head coil. MREG is a single-shot three-dimensional (3D) sequence that utilizes a spherical stack of spiral and undersamples 3D k-space trajectory (Zahneisen et al. 2012; Assländer et al. 2013; Lee et al. 2013). The following parameters were used for the 3D whole brain MREG sequence: repetition time (TR) = 100 ms, echo time (TE) = 36 ms, flip angle (FA) = 5°, field of view (FOV) = (192 mm)^3^, voxel size = 3 × 3 × 3 mm^3^. MREG data were reconstructed by L2-Tikhonov regularization with lambda = 0.1, with the latter regularization parameter determined by the L-curve method (Hugger et al. 2011), the resulting effective spatial resolution was 4.5 mm. MREG includes a dynamic off-resonance in k-space method, which corrects the respiration induced dynamic field-map changes in fMRI using 3D single shot techniques (Zahneisen et al. 2014). T1-weighted magnetization prepared rapid acquisition with gradient echo (MPRAGE) (TR = 1900 ms, TE = 2.49 ms, inversion time (TI) = 900 ms, FA 9°, FOV = 240, and slice thickness 0.9 mm) images were scanned for MREG data registration. During the 10-min resting-state scan, the subjects were instructed to lie still and awake in the scanner with their eyes open fixating on a cross on the screen. Soft pads were fitted over the study subjects’ ears to minimize motion and to protect hearing together with earplugs.

### Preprocessing

MREG data were preprocessed with Oxford Centre for Functional MRI of the Brain (FMRIB) software library (FSL) pipeline (Jenkinson et al. 2012) prior to single session ICA. The data were high-pass filtered with a cut-off frequency of 0.008 Hz (125 s). To minimize T1-relaxation effects, 180 time points were removed from the beginning of the data resulting in 5822 brain volumes in total. Motion correction was carried out using FSL MCFLIRT (Jenkinson et al. 2012), and all the data were visually inspected for spurious signal fluctuations. Brain extraction for 3D MPRAGE volumes was performed with FSL Brain Extraction TOOL (BET) using the following parameters: fractional intensity = 0.20–0.25, threshold gradient = 0.05–0.22, neck and bias-field correction. The extracted brain images were visually inspected to ensure optimal quality. Images were spatially smoothed with 5 mm full width and half maximum Gaussian kernel using “fslmaths.” MREG images were aligned to 3D (MPRAGE) anatomical images (full-search, 12 degree of freedom (DOF)) and to Montreal Neurological Institute (MNI 152) 4 mm^3^ standard space (full-search, 12 DOF) as a preprocessing step in FSL multivariate exploratory linear optimized decomposition into independent components (MELODIC) tool. Additionally, the advanced ICA FIX (FMRIBs ICA-based X-noisifier) method (Griffanti et al. 2014; Salimi-Khorshidi et al. 2014) was utilized to separate artifacts from neural signals in the rs-fMRI data. FIX was trained with previously collected control MREG data, and the applied FIX threshold was 10. The same FIX procedure was applied to each subject.

Global signal is thought to reflect physiological processes, motion, and other artifacts in addition to neuronal signal. However, global signal regression was not used in this study as the high temporal resolution, and thus critically sampled data, allows for discreet discrimination of cardiorespiratory signal from very low frequency signal (Huotari et al. 2019). Moreover, the advantages of global signal regression are still under debate (Murphy and Fox 2017).

### Lag Analysis Using DLA

**Figure 1:**
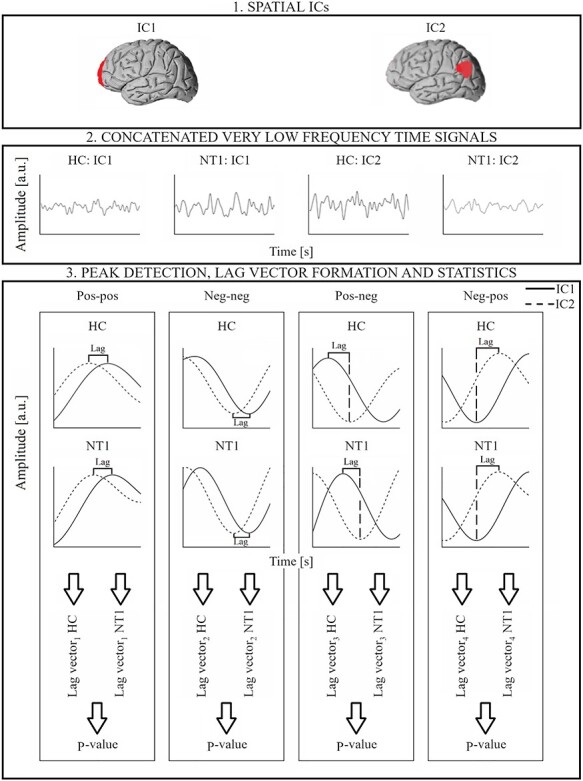
General workflow for DLA approach. (1) A given pair of RSN is selected. (2) Corresponding concatenated band-pass filtered (0.01–0.1 Hz) and detrended 10 min time signals for HC and NT1 groups. (3) For each of the 4 analysis (pos-pos, neg-neg, pos-neg, neg-pos), the lag values between the ICs are calculated, peak-by-peak, and separately for both groups. The corresponding *P*-value (for the given RSN pair) is calculated between the 2 lag vectors (HC and NT1).

A group level spatial ICA (multisession temporal concatenation in FSL) was performed for the FIX-cleaned data with a model order of 20 (*Z*-threshold = 2.3), NT1 and HC groups in the same group ICA analysis. As there are currently no RSN atlases of fast fMRI data available, we kept the FSL model order low to ease the RSN identification and to focus on the propagation sequencing between RSNs with the largest signal variance. In this work, the DLA approach was utilized for 4 separate analysis groups, that is, the time lags between RSNs were calculated (1) from positive to positive signal peak, (2) from negative to negative signal peak, (3) from positive to negative signal peak, and (4) from negative to positive signal peak (Fig. 1).

### Lag Between Activated RSNs (Between Positive Signal Peaks, pos-pos)

The same DLA workflow steps as described in a prior DLA paper (Raatikainen et al. 2019) were applied, with some improvements to the DLA method. (1) A pair of RSNs is selected. (2) The time signals were band-pass filtered to very low frequency band (0.01–0.1 Hz) and detrended, and each positive peak of the time signals was determined with the “findpeaks” function in MATLAB (“MinPeakDistance” output argument with the value of 100, that is, 10 s was used). Although the HC and NT1 groups have separate concatenated time signals, the filtering, detrending, and peak detection were applied separately for each subject individual time signals to avoid incorrect peaks in the signal discontinuities (as subject changes). (3) The lag vector was formed by calculating the time lag values between each positive peak (between RSNs) in the nearest neighbor principle (≤ ±5 s). In this study, the correlation versus anticorrelation was checked, that is, the signal phases (whether correlated or anticorrelated) of selected RSN pairs was checked for each peak pair. Therefore, the time lag was filled in the time lag vector only if the signals were in the same phase, that is, the positive peak of other RSN time signal was closer than the negative peak. Prior steps were completed separately for HC and NT1 data. Additional analysis parameters such as lag mean, median, and count values were calculated from the lag vectors. (4) The Kolmogorov–Smirnov test (“kstest2” in MATLAB) was calculated between HC and NT1 lag vectors to determine which RSN pairs had statistically significant differences in the lag patterns between HC and NT1 groups. All the steps (1–4) were completed separately for each selected RSN pair to construct the final *P*-value matrix.

### Lag Between Deactivated RSNs (Between Negative Signal Peaks, neg-neg)

The same DLA workflow steps (1–4) as described in the previous section were applied with the following exceptions. In step (2), each negative peak of the time signals was determined with “findpeaks” function in MATLAB. In the step (3), the lag vector was formed by calculating the time lag values between each negative peak (between RSNs) in the nearest neighbor principle (≤ ±5 s). Similarly, the signal phase (between RSNs) was checked, that is, the time lags of corresponding peaks were filled in the lag vector if the signals were in the same phase, that is, both peaks were negative.

### Lag From Activated RSN to Anticorrelated RSN (Between Positive and Negative Signal Peak, pos-neg)

Similarly, a given RSN pair was chosen in step (1), and the same filtering and detrending steps were applied as in the 2 previous sections. Here, in step (2) each positive peak of a reference time signal, and each negative peak of the other signal (time signal of other RSN in the RSN pair) was determined with “findpeaks” function in MATLAB. (3) The time lag value was calculated between the positive peak of the reference RSN and the next negative peak of other signal in the selected RSN pair. All the lags ≤ 5 s were filled in the corresponding lag vector. Prior steps were completed similarly for HC and NT1 data. (4) Kolmogorov–Smirnov test was calculated between HC and NT1 lag vectors. All steps were done separately for each RSN pair.

### Lag from Anticorrelated RSN to Activated RSN (Between Negative and Positive Signal Peak, neg-pos)

Similarly to the previous section, a given RSN pair was chosen (step 1) and the same filtering and detrending signal processing steps were applied (step 2). However, in step (2) each negative peak of the reference signal and each positive peak of the other signal in the given RSN pair were determined (“findpeaks” in MATLAB). (3) The time lag value was calculated between the negative peak of the reference RSN and the positive peak of the other signal in the selected RSN pair. All the lags ≤5 s were filled in the corresponding lag vector. Prior steps were completed similarly for HC and NT1 data, and finally, (4) Kolmogorov–Smirnov test was calculated between HC and NT1 lag vectors. All steps were completed separately for each RSN pair.

### Statistical Analysis

Surrogate data with identical preprocessing to the real data were created to evaluate the possibility of false positives in the *P*-value matrix values. Data consisted of 2 groups, both including 20 (equal to the RSN number) surrogate time signals (122 262 samples, i.e., the length of concatenated 10 min signals of 21 subjects) created with the “randi” function in MATLAB. The same processing steps introduced to real data were applied to surrogate time series including band-pass filtering to 0.01–0.1 Hz frequency band and signal detrending. Therefore, the spectral matching of the simulated data reflects the spectral content of postfiltered real data. The same DLA workflow steps as described in Raatikainen et al. (2019) were applied to the surrogate data (two-sided, two-sample Kolmogorov Smirnov test). The smallest *P*-value was determined as the threshold for significance. Since the lag values of antiphased signals were calculated separately for each direction of information flow, we decided to illustrate all the lag values as positive values in Figure 4. Finally, relative and absolute movement mean values were derived from FSL MCFLIRT and compared between both groups using *t*-tests.

## Results

### Summary

**Figure 2:**
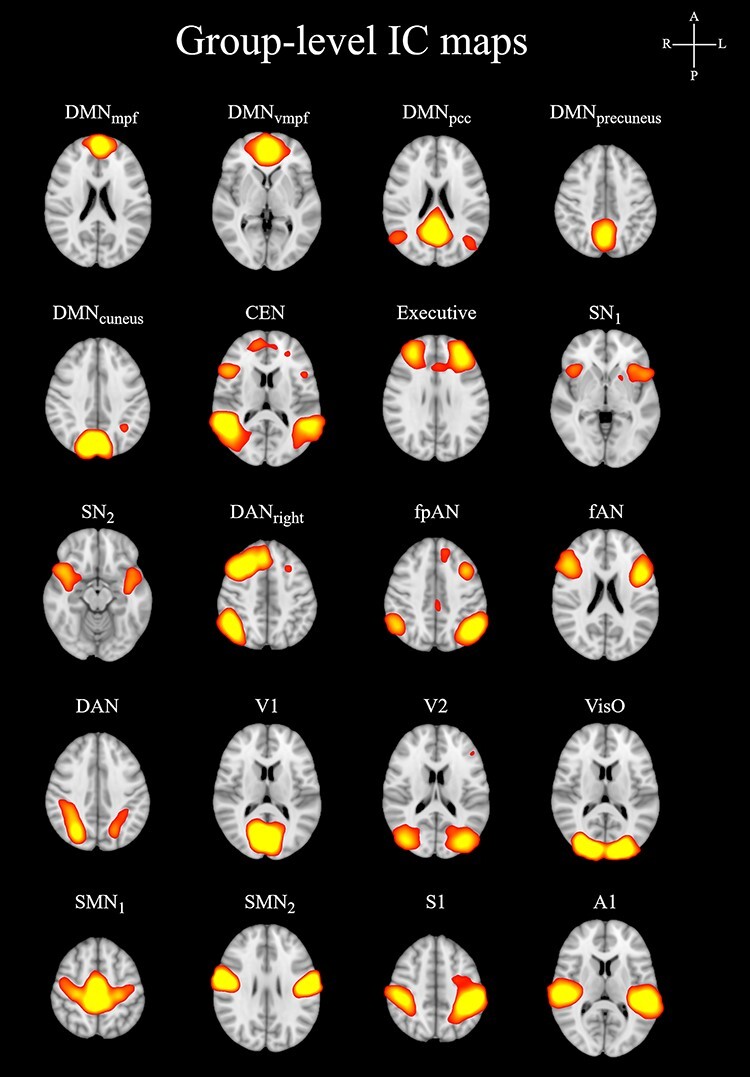
Group level IC maps. *Z*-value in the IC-maps is 3.

Twenty ICs were identified as RSN components by a neuroradiologist and used for further analysis (Fig. 2). The smallest *P*-value in the surrogate *P*-value matrix was 0.005, which was selected as a threshold for significance to reject the possibility of false positives and to declare statistically significant RSN combinations (*P*-value < 0.005; see Supplementary Fig. S1).

To summarize, of all statistically significant IC pairs (24), a pair with a DMN component was found in 20/24 (DMN_mpf_ (medial prefrontal): 3, DMN_vmpf_ (ventromedial prefrontal): 4, DMN_pcc_ (posterior cingulate cortex): 7, DMN_precuneus_: 3, DMN_cuneus_: 3), a pair with an executive component in 4/24 (CEN: 3, Executive: 1), a pair with a salience component in 5/24 (SN_1_: 3, SN_2_: 2), a pair with an attention component in 8/24 (DAN_Right_: 2, fpAN (frontoparietal attention network): 1, fAN (frontal attention network): 3, DAN: 2), a pair with a visual component in 8/24 (V1 (primary visual): 2, V2 (secondary visual): 0, VisO (visual occipital): 6) and a pair with A1 (primary auditory) in 3/24 pairs. The DMN group (DMN_mpf_, DMN_vmpf_, DMN_pcc_, DMN_precuneus_, DMN_cuneus_) has significant pairs with all other IC groups excluding sensorimotor group [SMN_1_ (sensorimotor network), SMN_2_, S1 (primary somatosensory)]. Four ICs were not included in any of the significant pairs: V2, SMN_1_, SMN_2_ and S1. The results were concentrated to the anticorrelative RSN pairs (20/24 of significant pairs). Interestingly, the DMN subcomponents did not form significant pairs with each other.

### Lag Distributions Between Activated (pos-pos) and Deactivated (neg-neg) RSNs

**Figure 3:**
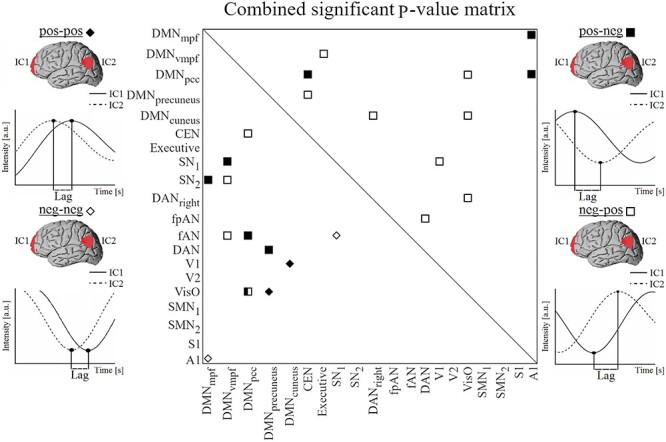
Combined significant (*P* < 0.005) *P*-value matrix for same phased (pos-pos marked with a black and neg-neg with a white diamond) and anticorrelated (pos-neg marked with a black and neg-pos with a white square) RSN pairs.

**Figure 4:**
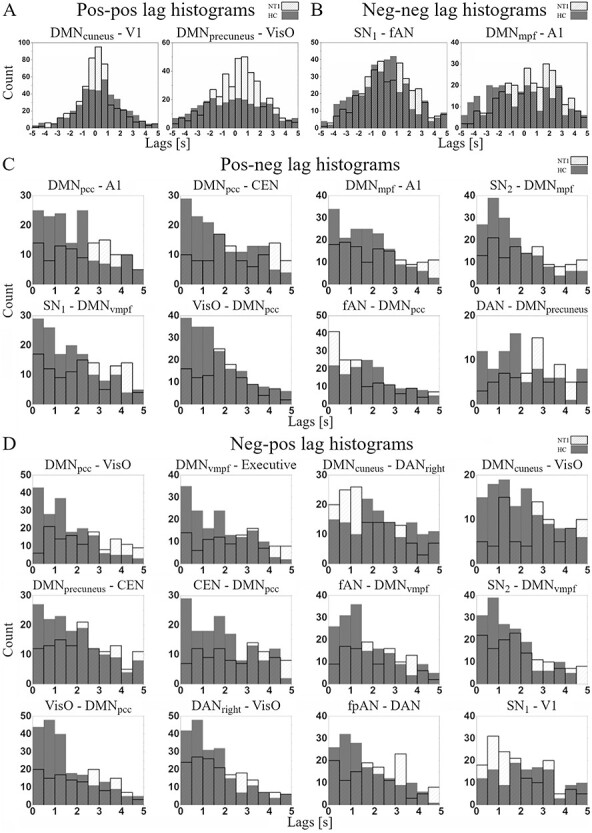
Lag value histograms of same phased (*A*) pos-pos, (*B*) neg-neg and anticorrelated (*C*) pos-neg, (*D*) neg-pos significant (*P* < 0.005) RSN pairs. HC group’s lag values are shown in dark gray and NT1 group’s lag values in white bins.

Two RSN-pairs survive the surrogate thresholding for significance in pos-pos (Fig. 3, Supplementary Fig. S2). A DMN subcomponent is included in both significant pairs: DMN_precuneus_ versus VisO and DMN_cuneus_ versus V1 (both at *P* = 0.001). In DMN_cuneus_ versus V1 the NT1 group has a high density of short lags around 0 s (median 0.1 s in NT1 and 0.4 s in HC, Fig. 4*A*, Supplementary Table S1). In DMN_precuneus_ versus VisO, the NT1 group’s lag distribution is skewed to the positive side (median 0.4 s in NT1 and −0.3 s in HC), suggesting a higher tendency of VisO being the source of information flow in NT1.

Two RSN-pairs survive the surrogate thresholding for significance in neg-neg (Fig. 3, Supplementary Fig. S3): DMN_mpf_ versus A1 and SN_1_ versus fAN (*P* < 0.001 and *P* = 0.004 respectively). In SN_1_ versus FAN, there is a higher tendency (median 0.3 s in NT1 vs. −0.1 s in HC, see Supplementary Table S2) that FAN deactivates before SN_1_ in NT1. In DMN_mpf_ versus A1, lag distribution of NT1 is skewed to the positive side reflected by the difference in medians (0.6 s in NT1 and −0.3 s in HC, Fig. 4*B*) and the %-ratio (39/60), suggesting that on average the deactivation of A1 precedes the deactivation of DMN_mpf_. The median value of −0.3 s suggests that DMN_mpf_ has a higher tendency to deactivate before A1 in HC.

### Lag Distributions in Anticorrelative RSNs (pos-neg and neg-pos)

Eight RSN-pairs survive the surrogate thresholding for significance in pos-neg (Fig. 3, Supplementary Fig. S4). A DMN subcomponent is included in all 8 significant pairs: DMN_mpf_ versus A1 (*P* = 0.005), DMN_pcc_ versus CEN (*P* = 0.001), DMN_pcc_ versus A1 (*P* = 0.001), SN_1_ versus DMN_vmpf_ (*P* = 0.005), SN_2_ versus DMN_mpf_ (*P* < 0.001), fAN versus DMN_pcc_ (*P* = 0.003), DAN versus DMN_precuneus_ (*P* = 0.001), and VisO versus DMN_pcc_ (*P* = 0.002).

In pos-neg lag histograms (Fig. 4*C*), the NT1 group has heterogenous lag distributions and a wide spread of lags. The HC group histograms appear slope-like with high density of lags close to 0 s, that is, shorter median (see Supplementary Table S3) values in 7/8 pairs suggesting more structured switching from activated RSN to deactivated RSN.

Twelve RSN-pairs survive the surrogate thresholding for significance in neg-pos (Fig. 3, Supplementary Fig. S5). 9/12 significant pairs include a DMN subcomponent: DMN_vmpf_ versus Executive (*P* < 0.001), DMN_pcc_ versus VisO (*P* < 0.001), DMN_precuneus_ versus CEN (*P* = 0.004), DMN_cuneus_ versus DAN_right_ (*P* = 0.003), DMN_cuneus_ versus VisO (*P* = 0.001), CEN versus DMN_pcc_ (*P* = 0.003), SN_2_ versus DMN_vmpf_ (*P* = 0.004), fAN versus DMN_vmpf_ (*p* = 0.004), and VisO vs. DMN_pcc_ (*p* < 0.001). Additionally, significant pairs include SN_1_ vs. V1 (*P* = 0.001), DAN_right_ versus VisO (*P* = 0.003), fpAN versus DAN (*P* = 0.004).

In neg-pos lag histograms, the NT1 group has heterogenous lag distributions with wide spread of lags (Fig. 4*D*). In comparison, the HC group has slope-like lag distributions with lag counts concentrated around 0 s, that is, shorter median values in 10/12 pairs (see Supplementary Table S4). These findings suggest that the switching from activated RSN to deactivated RSN and vice versa is aberrant in NT1.

### Motion

There were no statistically significant differences between HC and NT1 (see Supplementary Fig. S6) in mean absolute (*P* = 0.39) or relative movement (*P* = 0.34).

## Discussion

### Summary

In this study, we applied a recently described DLA approach (Raatikainen et al. 2019) together with fast fMRI MREG imaging to study lag patterns of intrinsic rs-fMRI signal in individuals with narcolepsy type 1 and healthy controls. Furthermore, we extended the analysis in a novel way to study lag pattern variations between RSNs in anticorrelating phases. To our knowledge, this is the first study to reveal fast fMRI information from the lag timings in inter-RSN activity state transitions, from deactivation to activation and from activation to deactivation, in the human brain. Notably, our results suggest that (1) DLA reveals abnormal inter-RSN propagation patterns in narcolepsy type 1 that manifest more robustly as alterations in the lag timings between activation and deactivation transitions (pos-neg and neg-pos, 83.3% of the significant pairs), (2) when considering the DMN and its relationship with other RSNs, information signaling is slower and monotonic in narcolepsy type 1 suggesting delayed transient coupling between these networks and that taken together (3) we consider that this slower flow of information between cognitively relevant RSNs may help to explain the deficits in sustained attention and executive function in narcolepsy type 1.

### RSN Interplay and Cognition

In our results, 83.3% of the significant altered pairs contained a DMN subcomponent. The high percentage of the DMN involvement with RSNs important in cognition (SN/DAN/CEN) and perception (V1, V2, VisO, A1) indicates that the changes in the dynamic inter-RSN connections concerning the DMN may be instrumental in narcolepsy type 1. This is further supported by proposed disease specific DMN finding in another rs-fMRI study (Drissi et al. 2016). In narcolepsy, sustained attention deficits have been observed (Fulda and Schulz 2001; Naumann and Daum 2003; Naumann et al. 2006; Witt et al. 2018). Interestingly, attentional impairments have been associated with damage to posterior cingulate cortex, a core node of the DMN, in the healthy brain (Bonnelle et al. 2011; Leech and Sharp 2014). Moreover, the changes in the DMN-involved connectivity within and between other networks are thought to underlie the sustained attention deficits present in schizophrenia, obsessive–compulsive disorder, and attention deficit hyperactivity disorder (Norman et al. 2017; Fan et al. 2018). Thus, our results suggest that the changes in connections concerning the DMN contribute to the prior reports of deficient sustained attention mechanism in narcolepsy type 1. Interestingly, we observed no significant pairs between the DMN subcomponents. This suggests that the robustness within the DMN is somewhat spared in narcolepsy type 1. However, the delayed and monotonic communication between the DMN and other RSNs, for example, the SN/DAN/CEN suggested by our results may lower the effect of these RSNs’ signaling to the DMN and manifest as a more erratic activation/deactivation behavior within the DMN.

Volume vice, the DMN is the largest RSN in our 20 IC model of fast fMRI splitting into 5 components. The high concentration of results to DMN could thus be confounded by the size of the DMN in comparison with other RSNs identified. However, if, for example, 4 of the attention related RSNs (DAN, DAN_right_, fpAN, and fAN forming a group comparable with the DMN in number of components, size, and spatial distribution to ventral and posterior components) were grouped and then compared with the DMN group, the DMN group would still overshadow the attention group (7/24 significant pairs in the attention group against 20/24 in the DMN group). Would the volume of the RSN have dominating effect on the results, then the 4 attention ICs should have approximately 16 significant pairs when the DMN components have 20. Furthermore, we observed no significant pairs between the DMN components—an improbable finding if the size of the components confounded the results. We suggest that rather our results reflect the changes in NT1, importance of the DNM, and its function as a hub.

Interactions between RSNs are crucial for complex cognition, as the DMN and SN/DAN/CEN interact to sustain normal cognitive function and attentional processes (Bressler and Menon 2010; Menon 2011; Uddin 2015; Chand et al. 2017). Our results show that in narcolepsy type 1, the reciprocal dynamic interplay between both the DMN/SN and DMN/DAN is delayed as illustrated by the lag distributions between the significant pairs including components of these RSNs. Moreover, our results show that the SN’s and DAN’s ability to suppress the DMN (in pos-neg DAN vs. DMN_precuneus_, SN_1_ and SN_2_ vs. DMN_vmpf_ and DMN_mpf_, respectively) may be decreased in narcolepsy type 1 compared with healthy controls. Additionally, the transient activation of the DMN after SN deactivation (in neg-pos SN_2_ vs. DMN_vmpf_) and the activation of the DAN following DMN deactivation (in neg-pos DMN_cuneus_ vs. DAN_right_) is atypical, indicating a failure to properly convey information. This may impair the SN’s and DAN’s modulation over the activity in the DMN and, moreover, the DMN’s modulation over the activity in the SN and DAN. To access attentional resources, the DMN, SN, and DAN form a causal hierarchically organized system in which the SN and DAN exert an inhibitory influence on the DMN and the DMN exerts an excitatory influence on the SN and DAN with the SN at the apex of the hierarchy modulating the other 2 anticorrelated networks (Zhou et al. 2018). In 2 rs-fMRI studies, Xiao et al. (2019, 2020) found hypoconnectivity between the DMN and SN/limbic system in narcolepsy type 1. Our results support findings of inter-RSN disruption between the DMN and SN. Moreover, the aberrant dynamic interplay between DMN and DAN may have a degenerative effect on sustained attention. The robustness of our results concerning attention is further strengthened by the fact that significant pairs between the DMN and other attention related RSNs (in pos-neg fAN vs. DMN_pcc_ and in neg-pos fAN vs. DMN_vmpf_) as well as between attention-related RSNs themselves and the SN (in neg-neg SN_1_ vs. fAN, in neg-pos fpAN vs. DAN) are present.

The slow information flow reflected by our lag results between the DMN and SN may additionally hinder the SN’s ability to switch between the DMN and CEN/Executive appropriately in narcolepsy type 1 compared with healthy controls. This may have an adverse effect on executive functions, as RSNs overlap with task-driven network identification providing a latent functional architecture readily engaged in the service of cognition (Smith et al. 2009; Laird et al. 2011; Spreng et al. 2013). Additionally, our results show that the information flow between the DMN and CEN/Executive may be bidirectionally compromised in narcolepsy type 1 (in pos-neg DMN_pcc_ vs. CEN and in neg-pos CEN vs. DMN_pcc_, DMN_precuneus_ vs. CEN and DMN_vmpf_ vs. Executive).

Interestingly, we observed no significant pairs between the sensorimotor group and other RSNs indicating fluent inter-RSN information signaling to and from sensorimotor associated spatial locations. Furthermore, this and the high involvement of the DMN in our results suggest that the lag related changes in narcolepsy type 1 are not completely global, but rather some inter-RSN connections are spared while others are affected.

The complete underlying mechanism of cognitive dysfunction in narcolepsy remains elusive, yet most prior research attributes the observed deficits to reduced efficiency of cognitive processing due to compensatory misallocation of available resources between different brain areas (Witt et al. 2018). Current information processing occurs simultaneously with the continuous monitoring and maintaining of alertness and attention in narcolepsy. In narcolepsy type 1, this is thought to relate to the changes in the vastly connected hypocretin system through labile cortical activation (Naumann et al. 2006). Our results indicate that in addition to misallocation of resources, relatively longer lags and thus slow information signaling between the RSN’s may contribute to the executive and attentional deficits in narcolepsy type 1. This is supported by the finding that alterations in brain connectivity have been associated with neuropsychological symptoms in narcolepsy type 1 (Xiao et al. 2020). The most probable explanation is that the hypoactivity of the hypocretin system underlies these lag-related dynamic changes.

### Resting-State Lag Analysis

Viewed from a spatiotemporal perspective, conventional zero-lag functional connectivity techniques have implicitly assumed that the involved spatial brain regions are at the same phase of a propagation pattern, thus leading to a simultaneous activation (Mitra and Raichle 2016). However, recently published findings have demonstrated that the rs-fMRI data are composed of multiple temporal sequences (Mitra, Snyder, Blazey, et al. 2015), where some regions are systematically early with respect to the rest of brain whereas others are systematically late (Mitra et al. 2014; Mitra, Snyder, Blazey, et al. 2015). However, these reference studies have assumed the existence of a single lag value over a given time epoch. Furthermore, the lag timings have been determined at a resolution finer than the temporal sampling frequency by parabolic interpolation.

More recent papers utilizing fast fMRI MREG imaging have estimated the inter-RSN lag timings dynamically between activated brain regions without cardiorespiratory aliasing and without need for data interpolation (Raatikainen et al. 2019). In this study, we further utilized the DLA method, and computed the mutual lag timings, peak-by-peak, not only between activated (positive peaks) and deactivated (negative peaks) RSNs but also between RSNs in their anticorrelating phases (from positive to negative peak and vice versa). Therefore, our study provides new insights relating to temporal dynamics of anticorrelated brain networks (negative associations between brain networks) their associated regions observed in the static studies (Fox et al. 2005, 2009; Uddin et al. 2009; Allen et al. 2011). Conversely, our results suggest that anticorrelative connections among brain networks are transient as opposed to constant across the whole scan, and this connectome is disrupted in narcolepsy type 1. Interestingly, the lag-pattern changes in our results are concentrated into the transiently anticorrelating RSN pairs (pos-neg, neg-pos: 83,3% of the significant pairs) over the transiently correlating RSN pairs (pos-pos, neg-neg: 16,7% of the significant pairs) indicating dysfunctionality in the transient activation/deactivation patterns in narcolepsy type 1 undetectable by conventional static zero-lag analyses. More generally, each RSN pair has mutual negative associations with each other for a specific temporal state and there are moments in which no negative associations exist. Our findings are in line with a recent study that investigated time-varying brain functional organizations (Iraji et al. 2019). They concluded that, anticorrelative relationships identified across previous default mode static analyses all exist, but in differing segments of time (Iraji et al. 2019). Therefore, our novel approach unveils typically overlooked features of brain dynamics and detects subtle alterations among patients with narcolepsy type 1 and potentially with other physiological or pathological conditions.

### Strengths and Limitations

All study patients were carefully clinically examined, and narcolepsy type 1 diagnosed according to criteria of ICSD third edition. The study groups were age- and sex-matched. High-temporal resolution of MREG sequence offered a high statistical power without the need for data interpolation in lag timings and enabled the removal of respiratory and cardiac peaks from the data. This enables accurate peak based lag pattern estimation. Rigorous motion control was utilized with visual inspection of the data, exclusion of subjects with excess motion and FIX. Additionally, no significant difference was observed between the study groups in relative and absolute motion.

Although all study subjects were instructed to stay eyes open in the scanner and the vigilance state was checked verbally between each scan, the propensity of narcolepsy patients to fall asleep could present a confounding factor in the lag estimation since the lag structure has been shown to alter between physiological states (Mitra, Snyder, Tagliazucchi, et al. 2015). However, most of the patients had medication that enhances vigilance and improves nighttime sleep quality. Since the NT1 group had heterogeneous drug combinations, it is difficult to evaluate the effect of medications in the lag metrics in this study, and due to ethical restrictions, we did not require the participants to refrain from medication prior to participating in this study. Therefore, supplementary analyses are needed in the future.

The potential hemodynamic response function (HRF) contribution to our between-group findings was not considered in our analyses. It has been recently shown that the interregional lags of BOLD fluctuations are reproducibly present in rs-fMRI data and are not attributable to hemodynamic factors (Mitra et al. 2014) and, instead, arise from neurophysiological origin (Lin et al. 2014; Mitra et al. 2014, 2018; Thompson et al. 2014; Rajna et al. 2015; Amemiya et al. 2016; Matsui et al. 2016; Vanni et al. 2017) However, a deeper understanding of potential HRF confounds are needed and thus the potential effects of HRF to lag dynamics should be considered in the future lag studies.

As this study was restricted to spatial topographies by the joined ICA analysis, it would be interesting to widen the lag metrics analysis to other brain regions relevant to narcolepsy type 1, for example, brain stem and basal ganglia. Moreover, a standardized cognition test battery would be important in a more thorough evaluation of the association of cognition and lag pattern variations in narcolepsy type 1. Finally, investigation of the relationship between DLA-based lag values and correlation values would be interesting in the future. All of the above are important issues that should be studied in a more detailed manner in the future and with larger study population.

## Conclusion

Understanding the neurobiology of narcolepsy type 1 requires a critical investigation of brain temporal dynamics. Our results reveal delayed information flow prominently between the DMN and other major RSNs in narcolepsy type 1. Our results add to the evidence of an imbalance in the intrinsic RSN connectivity in narcolepsy type 1, and help to explain the deficits in sustained attention and executive function observed in the earlier studies. This study addresses an essential question regarding the extent and nature of dynamic time lag variations between brain regions by utilizing both fast fMRI imaging and DLA. Our findings are novel, as the dynamic internetwork lag variations were investigated, peak-by-peak, both between activated and deactivated RSNs, and additionally, between anticorrelated networks in critically sampled data. Finally, this work challenges the notion that anticorrelative connections to brain networks are constant over the entire scan, and highlights the importance of exploring inter-RSN phase transitions as transient events.

## Supplementary Material

Supplementary_figureS1_tgaa073

Supplementary_figureS2_tgaa073

Supplementary_figureS3_tgaa073

Supplementary_figureS4_tgaa073

Supplementary_figureS5_tgaa073

Supplementary_figureS6_tgaa073

Supplementary_tables_and_figure_captions_tgaa073

## Notes

Thanks to Instrumentarium Science Foundation, North Ostrobothnia Regional Fund, Finnish Brain Foundation sr, Finnish Epilepsy Association, Finnish Medical Foundation, Maire Taponen Foundation, Orion Research Foundation, Walter Ahlström Foundation, Finnish Foundation for Technology Promotion, Tauno Tönning Foundation, Radiological Society of Finland, The University of Oulu Scholarship Foundation, Emil Aaltonen Foundation, and Medical Research Center Oulu who supported this research by a personal grant. We thank all the study participants whose attendance made this study possible. Finally, we wish to acknowledge CSC — IT Center for Science, Finland, for computational resources. *Conflict of Interest*: None declared.
