# Supplementary figures and images for: Lag Analysis of Fast fMRI Reveals Delayed Information Flow Between the Default Mode and Other Networks in Narcolepsy
Source: Cereb Cortex Commun. 2020 Oct 10;1(1):tgaa073. doi: 10.1093/texcom/tgaa073 (PMC8153076; doi:10.1093/texcom/tgaa073)

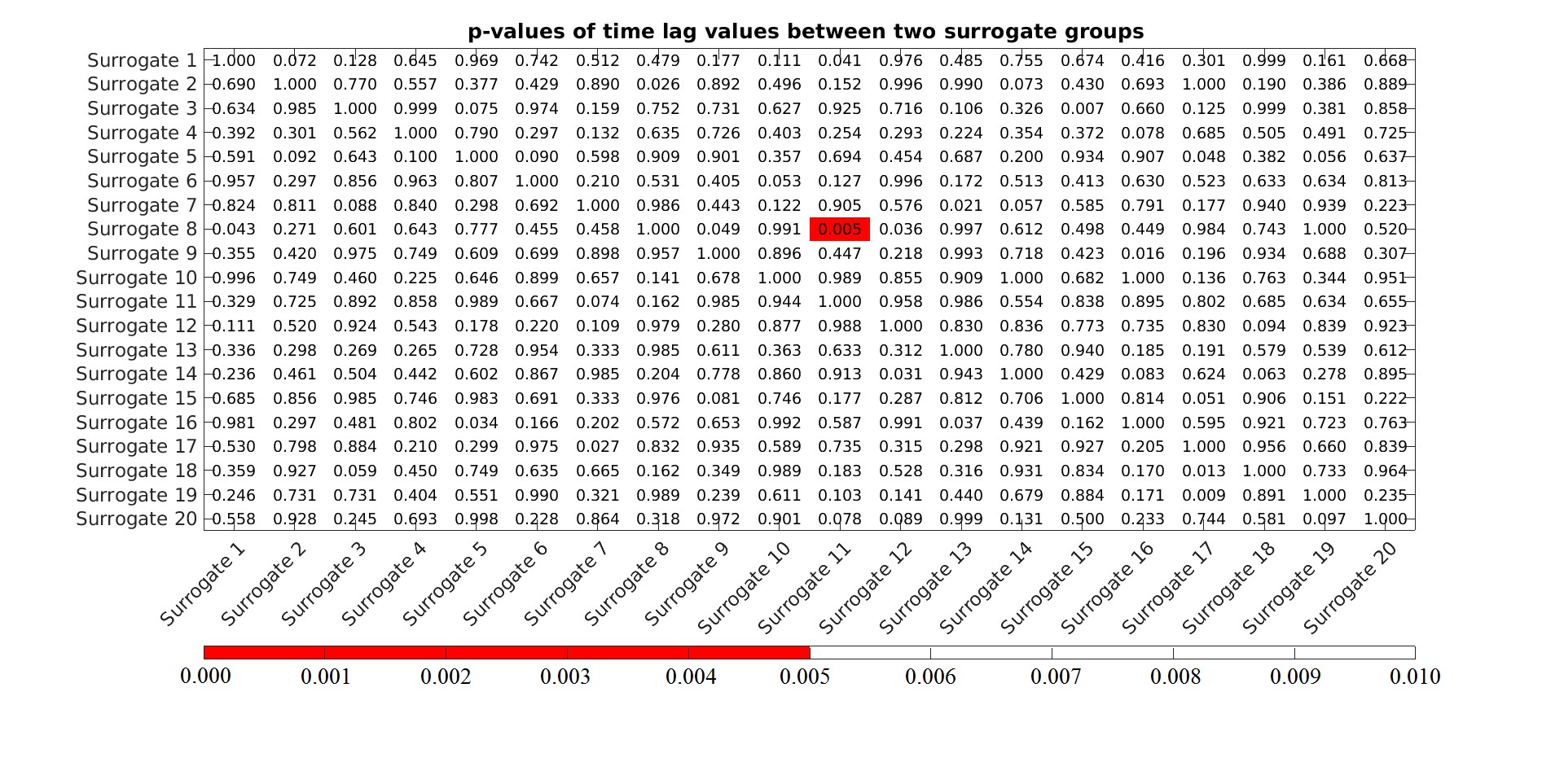

Supplement: Supplementary_figureS1_tgaa073 [file supplementary_figures1_tgaa073.jpeg]

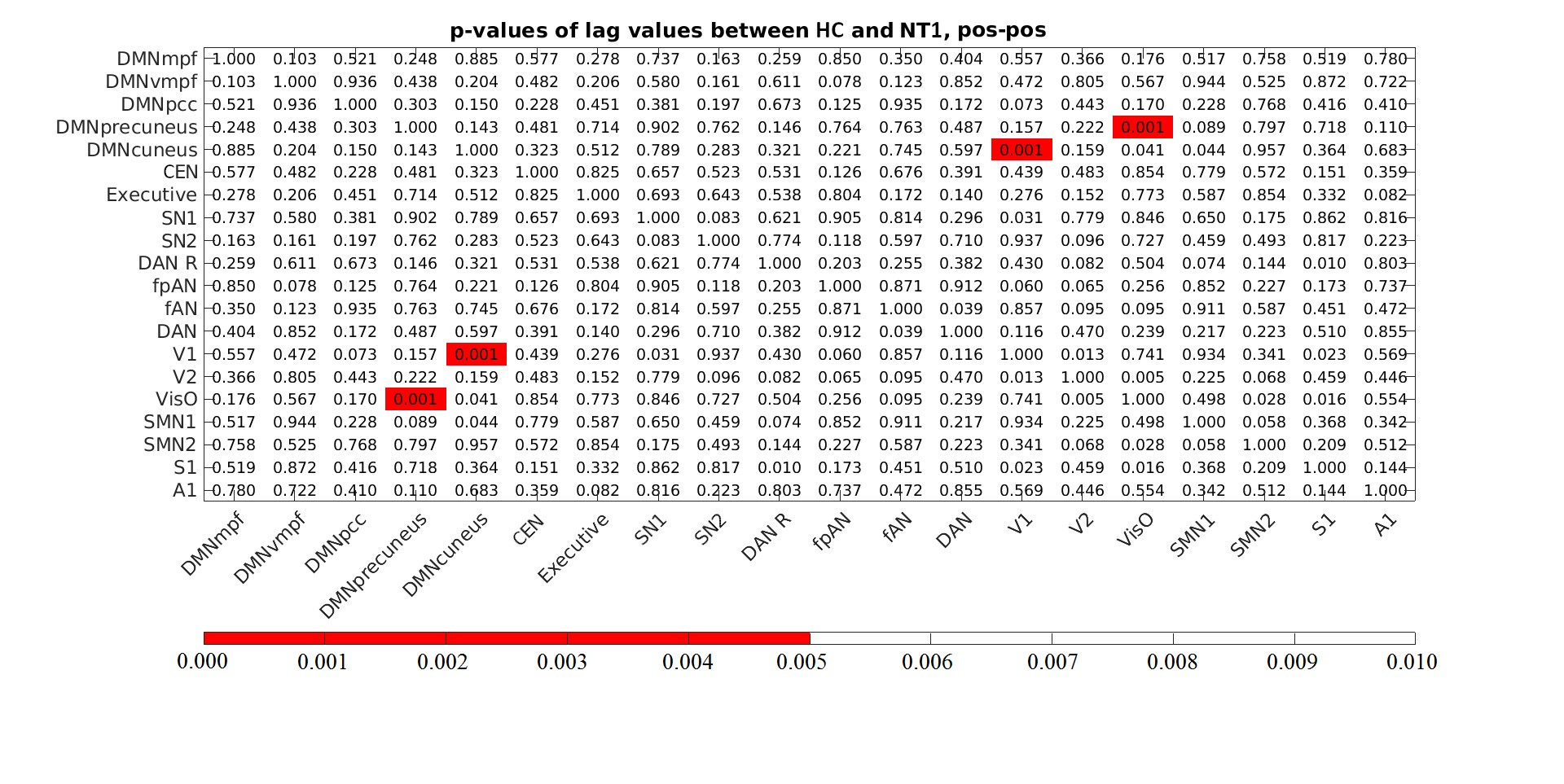

Supplement: Supplementary_figureS2_tgaa073 [file supplementary_figures2_tgaa073.jpeg]

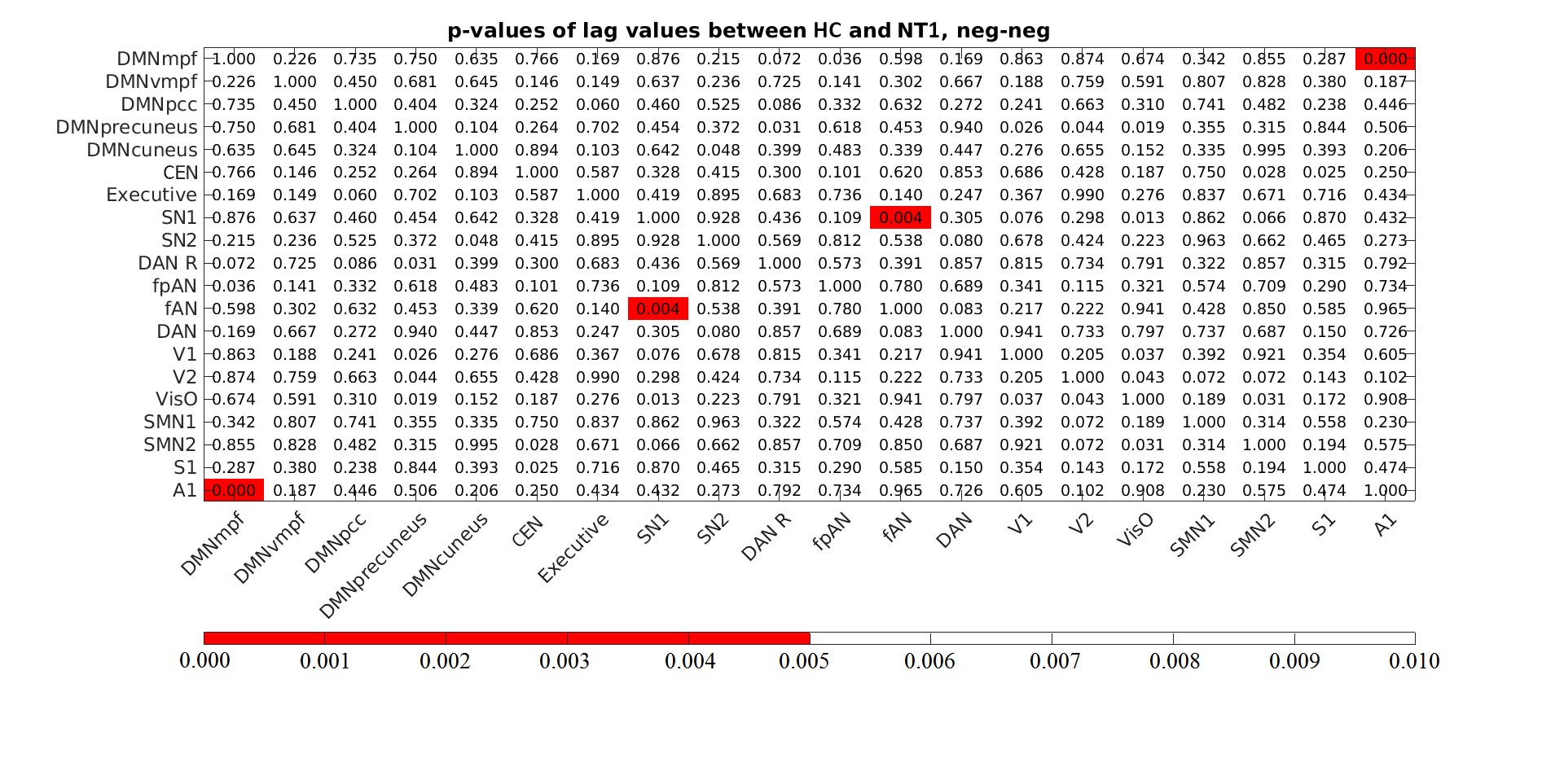

Supplement: Supplementary_figureS3_tgaa073 [file supplementary_figures3_tgaa073.jpeg]

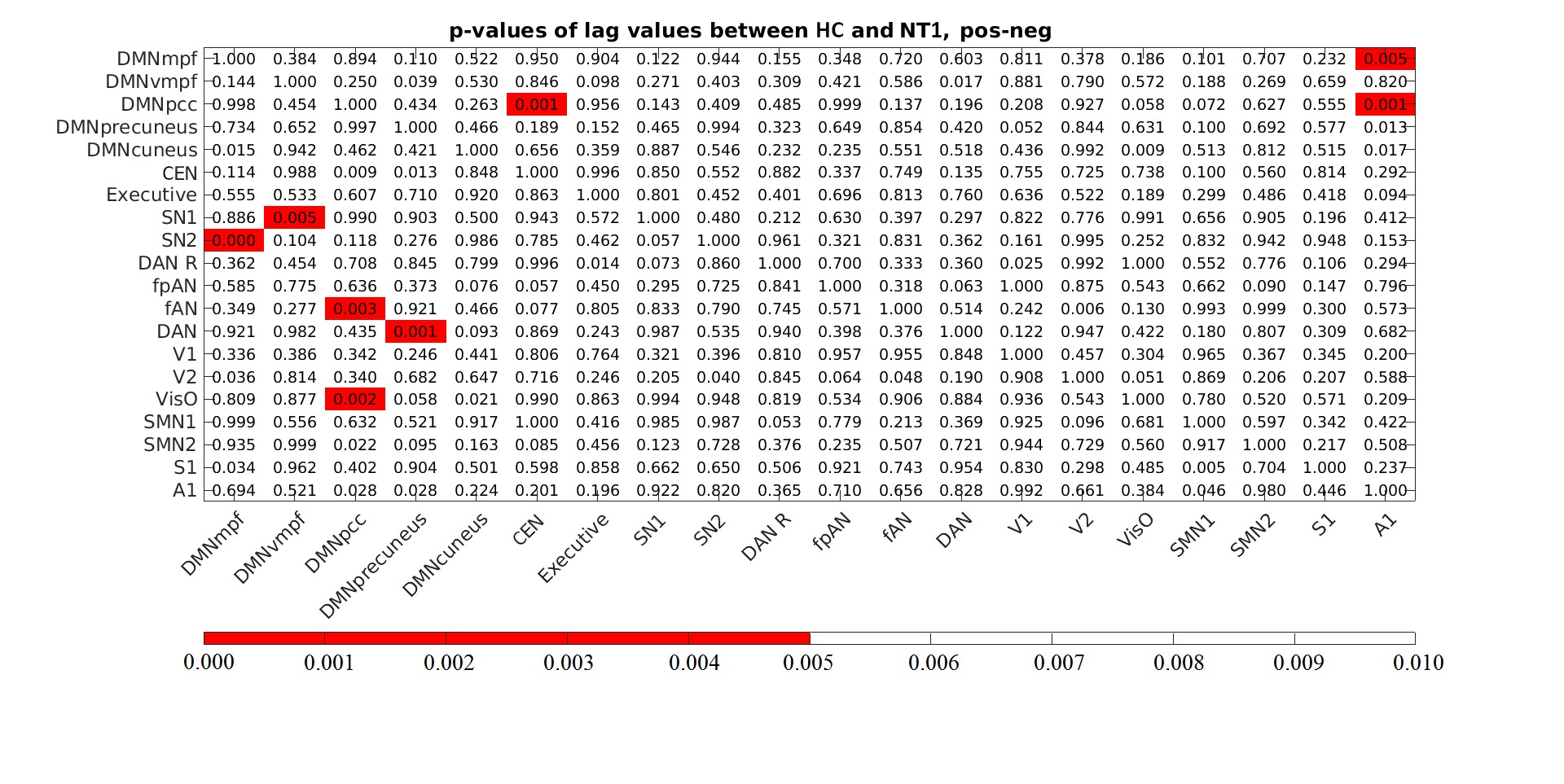

Supplement: Supplementary_figureS4_tgaa073 [file supplementary_figures4_tgaa073.jpeg]

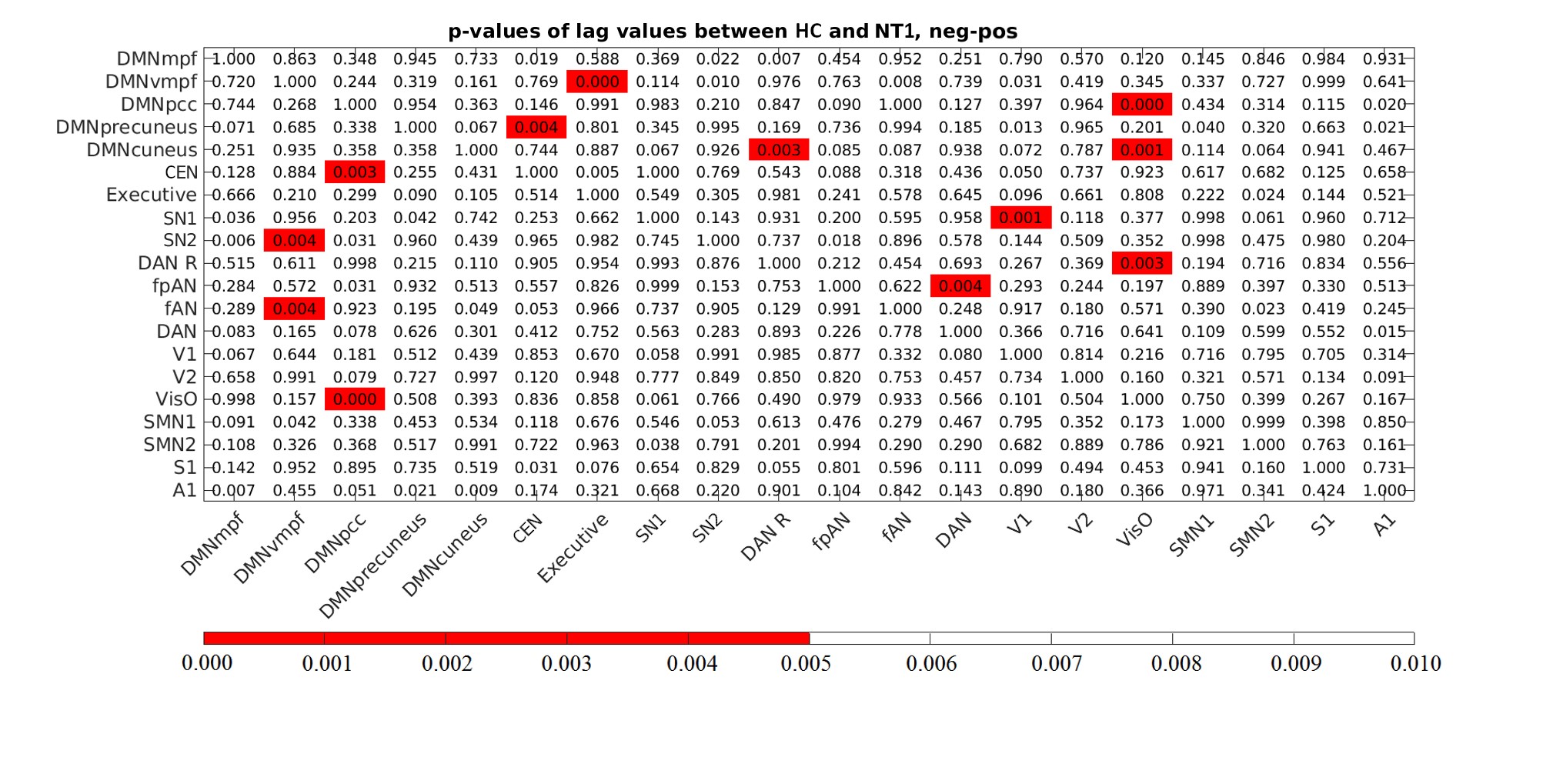

Supplement: Supplementary_figureS5_tgaa073 [file supplementary_figures5_tgaa073.jpeg]

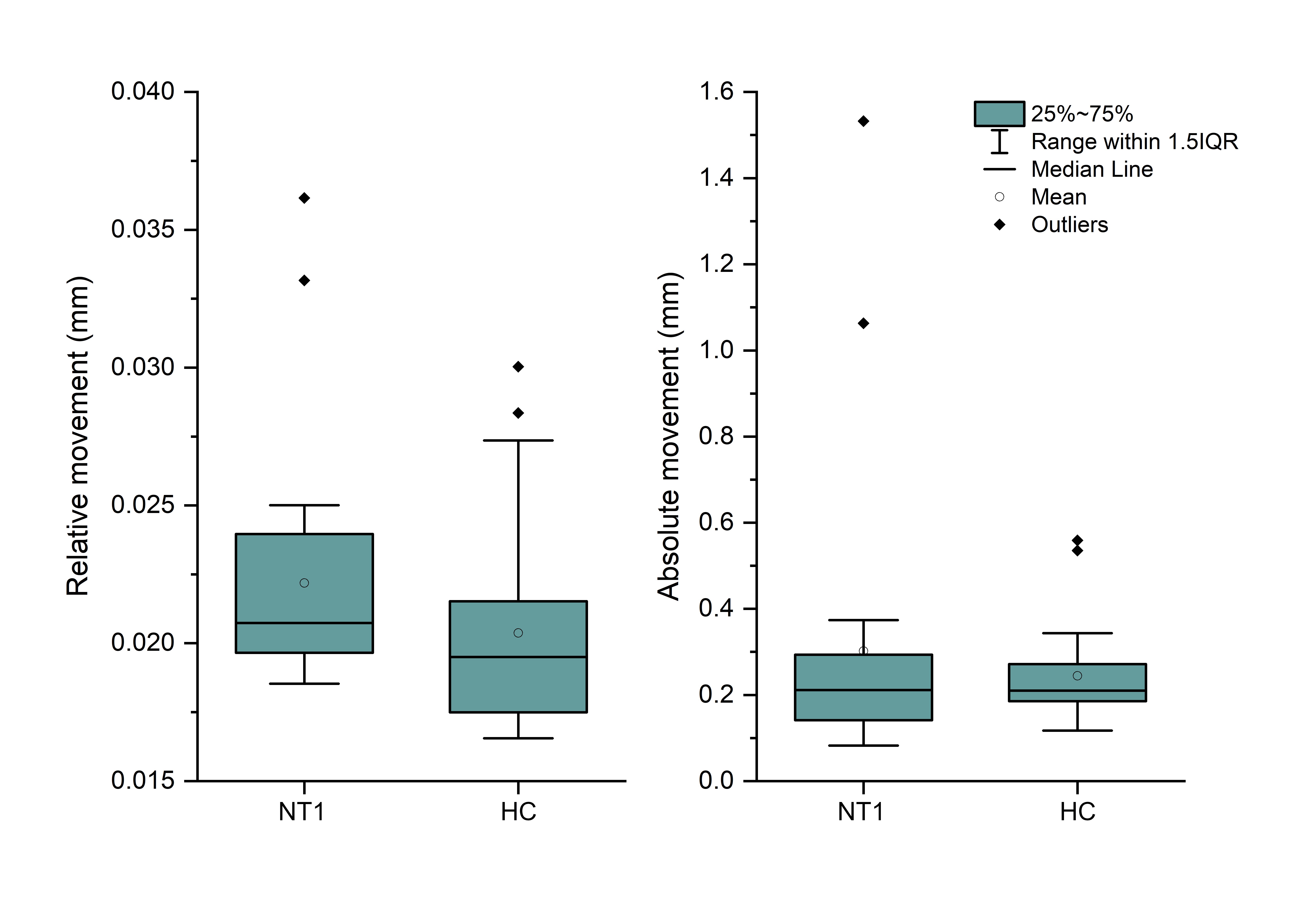

Supplement: Supplementary_figureS6_tgaa073 [file supplementary_figures6_tgaa073.jpeg]
