# Supplementary material for: Lag Analysis of Fast fMRI Reveals Delayed Information Flow Between the Default Mode and Other Networks in Narcolepsy
Source: Cereb Cortex Commun. 2020 Oct 10;1(1):tgaa073. doi: 10.1093/texcom/tgaa073 (PMC8153076; doi:10.1093/texcom/tgaa073)
Supplement: Supplementary_tables_and_figure_captions_tgaa073 [file supplementary_tables_and_figure_captions_tgaa073.docx]

**Supplementary Material**

**Tables**

**Supplementary Table S1**.

| **POS - POS** | | | | | | | | | |
| --- | --- | --- | --- | --- | --- | --- | --- | --- | --- |
|  |  | HC | |  |  |  | NT1 | |  |
|  | Counts | Mean | Median | %-ratio |  | Counts | Mean | Median | %-ratio |
| DMN_prec_ - VisO | 285 | -0.23 | -0.3 | 52/46 |  | 408 | 0.15 | 0.4 | 42/56 |
| DMN_cuneus_ - V1 | 427 | 0.31 | 0.4 | 41/57 |  | 500 | 0.12 | 0.1 | 44/53 |
| ^[[1]](#footnote-1)^ | | | | | | | | | |

**Supplementary Table S2**.

| **NEG - NEG** | | | | | | | | | |
| --- | --- | --- | --- | --- | --- | --- | --- | --- | --- |
|  |  | HC | |  |  |  | NT1 | |  |
|  | Counts | Mean | Median | %-ratio |  | Counts | Mean | Median | %-ratio |
| DMN_mpf_ - A1 | 269 | -0.23 | -0.3 | 52/47 |  | 296 | 0.5 | 0.6 | 39/60 |
| SN_1_ - fAN | 373 | -0.12 | -0.1 | 51/48 |  | 377 | 0.29 | 0.3 | 46/53 |
| ^[[2]](#footnote-2)^ | | | | | | | | | |

**Supplementary Table S3.**

| **POS - NEG** | | | | | | | |
| --- | --- | --- | --- | --- | --- | --- | --- |
|  | HC | | |  | NT1 | | |
|  | Counts | Mean (s) | Median (s) |  | Counts | Mean (s) | Median (s) |
| DMN_mpf_ - A1 | 170 | 1.76 | 1.75 |  | 136 | 2.24 | 2.1 |
| DMN_pcc_ - A1 | 147 | 1.85 | 1.6 |  | 110 | 2.39 | 2.4 |
| DMN_pcc_ - CEN | 147 | 1.84 | 1.6 |  | 102 | 2.53 | 2.35 |
| SN1 - DMN_vmpf_ | 146 | 1.78 | 1.6 |  | 114 | 2.3 | 2.25 |
| SN2 - DMN_mpf_ | 168 | 1.66 | 1.3 |  | 131 | 2.28 | 2.2 |
| fAN - DMN_pcc_ | 148 | 1.98 | 1.8 |  | 150 | 1.66 | 1.3 |
| DAN - DMN_precuneus_ | 82 | 2.1 | 1.85 |  | 67 | 2.68 | 2.8 |
| VisO - DMN_pcc_ | 194 | 1.68 | 1.4 |  | 118 | 1.98 | 1.85 |
| ^[[3]](#footnote-3)^ | | | | | | | |

**Supplementary Table S4.**

| **NEG - POS** | | | | | | | |
| --- | --- | --- | --- | --- | --- | --- | --- |
|  | HC | | |  | NT1 | | |
|  | Counts | Mean (s) | Median (s) |  | Counts | Mean (s) | Median (s) |
| DMN_vmpf_ - Executive | 163 | 1.64 | 1.5 |  | 106 | 2.42 | 2.5 |
| DMN_pcc_ - VisO | 193 | 1.47 | 1.3 |  | 130 | 2.38 | 2.4 |
| DMN_precuneus_ - CEN | 155 | 1.86 | 1.6 |  | 127 | 2.36 | 2.3 |
| DMN_cuneus_ - DAN_right_ | 140 | 2.33 | 2.3 |  | 147 | 1.87 | 1.5 |
| DMN_cuneus_ - VisO | 132 | 1.98 | 1.7 |  | 80 | 2.69 | 2.8 |
| CEN - DMN_pcc_ | 156 | 1.82 | 1.65 |  | 97 | 2.55 | 2.6 |
| SN_2_ - DMN_vmpf_ | 175 | 1.46 | 1.2 |  | 141 | 1.98 | 1.9 |
| fAN - DMN_vmpf_ | 172 | 1.69 | 1.4 |  | 120 | 2.11 | 1.9 |
| VisO – DMN_pcc_ | 216 | 1.51 | 1.2 |  | 133 | 2.1 | 2 |
| SN1 - V1 | 129 | 2.3 | 2.2 |  | 159 | 1.84 | 1.6 |
| DAN_right_ - VisO | 211 | 1.46 | 1.1 |  | 171 | 1.87 | 1.7 |
| fpAN - DAN | 159 | 1.65 | 1.4 |  | 135 | 2.1 | 2 |
| ^[[4]](#footnote-4)^ | | | | | | | |

**Supplementary figure captions**

**Supplementary Fig. S1.** p-value matrix between two surrogate groups. The smallest value was selected as a threshold for significance (p < 0.005).

**Supplementary Fig. S2.** p-value matrix of the significant (p < 0.005) pairs in pos-pos.

**Supplementary Fig. S3.** p-value matrix of the significant (p < 0.005) pairs in neg-neg.

**Supplementary Fig. S4.** p-value matrix of the significant (p < 0.005) pairs in pos-neg.

**Supplementary Fig. S5.** p-value matrix of the significant (p < 0.005) pairs in neg-pos.

**Supplementary Fig. S6.** Relative and absolute movement values between narcolepsy (NT1) and control group (HC) in mm.

1. Lag counts, mean, median and %-ratio (how often the former RSN is leading vs. the latter) in significant RSN pairs in pos-pos between the narcolepsy group (NT1) and healthy control group (HC). Prec = precuneus. [↑](#footnote-ref-1)
2. Lag counts, mean, median and %-ratio (how often the former RSN is leading vs. the latter) in significant RSN pairs in neg-neg between the healthy control group (HC) and the narcolepsy group (NT1). [↑](#footnote-ref-2)
3. Lag counts, mean and median in significant RSN pairs in pos-neg between the healthy control (HC) and narcolepsy groups (NT1). [↑](#footnote-ref-3)
4. Lag counts, mean and median in significant RSN pairs in neg-pos between the healthy control (HC) and narcolepsy groups (NT1). [↑](#footnote-ref-4)
